# Supplementary material for: Skipping Breakfast and a Meal at School: Its Correlates in Adiposity Context. Report from the ABC of Healthy Eating Study of Polish Teenagers
Source: Nutrients. 2019 Jul 11;11(7):1563. doi: 10.3390/nu11071563 (PMC6682891; doi:10.3390/nu11071563)
Supplement: Supplementary file 1 [file nutrients-11-01563-s001.pdf]

Skipping breakfast and a meal at school: its correlates in adiposity context. Report from the ABC of Healthy Eating study of Polish teenagers

Lidia Wadolowska, Jadwiga Hamulka, Joanna Kowalkowska, Natalia Wojtas, Magdalena Gornicka, Marta Jeruszka-Bielak, Malgorzata Kostecka, Agata Wawrzyniak

Supplementary Materials

Table S1. Questions and correct answers (scored with 1 point) regarding an assessment of nutrition knowledge and percentage of participants with correct answers.

No.	Question	Correct answer	Participants with correct answers (%)
1	The key to healthy eating is:	All answers: to eat many different kinds of foods, to eat more of some products than others and to eat moderate or small amounts of some products.	17.4
2	How much of milk and milk beverages, e.g., kefir, yoghurt, should you eat during a day?	Two glasses.	21.7
3	How much fruits and vegetables should you eat?	Five or more fruits and vegetables a day.	27.4
4	"Good" microorganisms are those microorganisms which:	All answers: cause food fermentation, cause bread rising and have positive influence on human health.	7.6
5	Fast foods contain much of:	Fat and salt.	71.8
6	Which set of products contains much fibre?	Whole-meal bread, apple, bean.	46.1
7	Main sources of calcium in diet are:	Dairy products.	40.1
8	Which breakfast set contains less fat?	Corn flakes with full fat milk.	9.5
9	Main function of protein in the body is:	Regulation and structure.	14.8
10	Alkalizing products are:	Fruits, vegetables and potatoes.	9.4
11	Which information on the food label is most important for every consumer's food safety?	Shelf life.	34.6
12	Vegan diet means that one is eliminating from diet:	Meat, dairy and eggs.	35.7
13	Amount of human energy requirement depends on:	Age, sex and physical activity.	48.7
14	BMI is a ratio of:	Body weight and height.	21.6
15	To be active means:	All answers: go to the gym, walk often and play sports e.g. football, volleyball, bike riding.	53.5
16	Which way of cooking is the best for vitamin C retention in potatoes?	Steam cooking.	24.3
17	Where one should keep an open container of juice:	In a refrigerator.	75.8
18	Are energy drinks safe for adolescents?	Should not be consumed before age of 16.	47.8

Table S2. Scoring (with points) of answers describing the characteristics of the household affluence to determine Family Affluence Scale (range: 0–7 points).

Questions		Answers scored with			
		0 points	1 point	2 points	2 points
1.	'Does your family own a car, van or truck?'	No	Yes, one	Yes, two or more	

2.	'During the past year, how many times did you travel away on holiday with your family?'	Not at all	Once	Twice	More than twice
3.	'Do you have your own bedroom for yourself?'	No	Yes		
4.	'How many computers or laptops or tablets does your family own?'	None	One	Two	More than two

Notes: For all questions, necessary examples and explanations were given.

Table S3. Distribution of subjects skipping breakfast and a meal at school ($p < 0.0001$) (percentages of the total sample).

Characteristics	Skipping meal at school (number of school days/week)		
	0/week (n=1078)	1-2/week (n=284)	3-5/week (n=201)
Skipping breakfast (number of days/week)			
0/week (n=1095)	51.9	11.4	6.7
1-3/week (n=195)	7.2	3.6	1.7
4-7/week (n=273)	9.8	3.2	4.5

Notes: n: sample size; Sample size (n=1563) is lower (by 3 subjects) than total sample due to missing data.

Table S4. Meal-adjusted association of skipping breakfast or a meal at school with socioeconomic correlates, nutrition knowledge and lifestyle in teenagers. (Meal-adjusted odds ratios and 95% confidence intervals; multivariate models).

Characteristics	Skipping breakfast (referent: never)				Skipping a meal at school (referent: never)			
	A Few Times a Week		Frequently		A Few times a Week		Frequently	
Girls (ref.: boys)	1.63**	1.18, 2.26	1.55**	1.16, 2.06	0.66**	0.50, 0.86	0.69*	0.50, 0.96
Age (years)								
12 (ref.: 11)	1.01	0.64, 1.61	1.14	0.77, 1.68	1.11	0.76, 1.63	1.03	0.66, 1.60
13 (ref.: 11)	1.27	0.67, 2.40	1.78*	1.00, 3.18	1.20	0.68, 2.12	1.65	0.88, 3.10
Urban residence (ref.: rural)	0.81	0.59, 1.11	1.00	0.88, 1.14	0.80	0.61, 1.05	1.41*	1.01, 1.97
Family Affluence Scale								
moderate (ref.: high)	1.60*	1.06, 2.41	1.58*	1.08, 2.30	1.20	0.85, 1.69	0.96	0.64, 1.44
low (ref.: high)	1.80*	1.12, 2.89	2.48****	1.64, 3.74	1.20	0.80, 1.81	1.20	0.76, 1.89
Nutrition knowledge score								
moderately-low (ref.: higher)	1.28	0.86, 1.89	1.02	0.71, 1.46	0.97	0.70, 1.34	1.94**	1.25, 3.00
lowest (ref.: higher)	1.32	0.86, 2.03	1.41	0.97, 2.03	1.01	0.72, 1.40	2.05**	1.31, 3.23
Physical activity level								
moderate (ref.: high)	1.42	0.97, 2.06	0.88	0.64, 1.21	1.65**	1.20, 2.26	1.58*	1.08, 2.31
low (ref.: high)	1.20	0.63, 2.28	1.24	0.75, 2.03	1.90*	1.15, 3.15	1.94*	1.08, 3.48
Screen time (hours/day)								
2 to <4 (ref.: <2)	1.75**	1.23, 2.49	1.78***	1.28, 2.46	1.36*	1.01, 1.84	1.01	0.67, 1.53
≥4 (ref.: <2)	1.78*	1.14, 2.77	2.56****	1.76, 3.72	1.36	0.93, 1.99	2.10***	1.41, 3.12
Moderate/high pHDI (ref.: low)	0.56**	0.38, 0.82	0.98	0.70, 1.35	0.77	0.57, 1.05	0.68*	0.46, 0.99
Moderate/high nHDI (ref.: low)	0.39*	0.16, 0.93	1.21	0.73, 2.01	0.67	0.36, 1.26	1.06	0.59, 1.89

Notes: Sample size may vary in variables due to missing data; Confounders included in meal-adjusted odds ratio: gender, age (years), residence (categorical variable), Family Affluence Scale (points), nutrition knowledge (points), physical activity (categorical variable), screen time (categorical variable) and consumption of breakfast or a meal at school (categorical variables), excluding the modelled variable from the confounders set, respectively; Categories of FAS: low (0-4 points), moderate (5-6 points), high (7 points); Categories of nutrition knowledge score: the lowest (0-4 points), moderately-low (5-7 points), higher (8-18 points); Categorizing of physical activity was based on data regarding to physical activity at school and leisure time – details are given in Table 2; pHDI: pro-Healthy Diet Index; nHDI: non-Healthy Diet Index; Categories of pHDI and nHDI: low (<33.33% points), moderate/high (≥33.33% points); Skipping meals: 'never' – consumption of breakfast 7 days/week, consumption of a meal at school 5 school days/week, 'a few times a week' – consumption of breakfast 4-6 days/week, consumption of a meal at school 3-4 school days/week, 'frequently' – consumption of breakfast 0-3 days/week, consumption of a meal at school 0-2 school days/week; Statistically significant (Wald's statistics): * $p < 0.05$; ** $p < 0.01$; *** $p < 0.001$; **** $p < 0.0001$; ns: not statistically significant.

Table S5. Meal-adjusted association of skipping breakfast or a meal at school with adiposity markers in teenagers. (Meal-adjusted odds ratios and 95% confidence intervals; multivariate models).

Characteristics	BMI-for-age categories[a] (ref.: normal)				Central obesity[b] (ref.: lack)	
	thinness		overweight/obesity			
Skipping breakfast						
Never	1.00		1.00		1.00	
A Few Times a Week	0.47*	0.24, 0.93	1.21	0.83, 1.76	1.69*	1.06, 2.70
Frequently	0.54*	0.29, 0.98	1.87***	1.36, 2.56	1.73**	1.15, 2.60
Skipping a meal at school						
Never	1.00		1.00		1.00	
A Few Times a Week	0.88	0.63, 1.24	1.15	0.83, 1.60	0.81	0.53, 1.24
Frequently	0.87	0.58, 1.30	1.12	0.77, 1.63	0.79	0.48, 1.30

Notes: Sample size may vary in variables due to missing data; Confounders included in meal-adjusted odds ratio: gender, age (years), residence (categorical variable), Family Affluence Scale (points), nutrition knowledge (points), physical activity (categorical variable), screen time (categorical variable) and consumption of breakfast or a meal at school (categorical variables), excluding the modelled variable from the confounders set, respectively; Skipping meals: 'never' – consumption of breakfast 7 days/week, consumption of a meal at school 5 school days/week, 'a few times a week' – consumption of breakfast 4-6 days/week, consumption of a meal at school 3-4 school days/week, 'frequently' – consumption of breakfast 0-3 days/week, consumption of a meal at school 0-2 school days/week; BMI: Body mass index; [a]BMI-for-age categorised according to gender-specific BMI cut-offs for teenagers [44]: thinness BMI<18.5 kg/m^2; normal weight BMI=18.5 to 24.9 kg/m^2; overweight/obesity BMI≥25 kg/m^2; [b]Central obesity identified as waist-to-height ratio ≥0.5 according to Ashwell *et al*. [18]; Statistically significant (Wald's statistics): * $p < 0.05$; ** $p < 0.01$; *** $p < 0.001$; **** $p < 0.0001$.
